# Filgrastim XM02 (Tevagrastim®) after autologous stem cell transplantation compared to lenograstim: favourable cost-efficacy analysis

**DOI:** 10.3332/ecancer.2013.327

**Published:** 2013-06-25

**Authors:** A Gardellini, F Gigli, A Babic, G Andreola, D Radice, S Sammassimo, G Martinelli, D Laszlo

**Affiliations:** 1 Division of Haematoncology, European Institute of Oncology, Milan, Italy; 2 Division of Statistics, European Institute of Oncology, Milan, Italy; 3 Stem Cell Collection Unit, European Institute of Oncology, Milan, Italy

**Keywords:** biosimilar G-CSF, autologous bone marrow transplantation, engraftment, filgrastim, lenograstim

## Abstract

**Purpose:**

Granulocyte colony-stimulating factors (G-CSFs), filgrastim and lenograstim, are recognised to be useful in accelerating engraftment after autologous stem cell transplantation. Several forms of biosimilar non-glycosylated G-CSF have been approved by the European Medicines Agency, with limited published data supporting the clinical equivalence in peripheral blood stem cell mobilisation and recovery after autologous stem cell transplantation.

**Method:**

With the aim of comparing cost-effective strategies in the use of G-CSF after autologous stem cell transplantation, we retrospectively evaluated 32 patients consecutively treated with biosimilar filgrastim XM02 (Tevagrastim) and 26 with lenograstim. All patients received G-CSF (biosimilar or lenograstim) at a dosage of 5 mcg/kg/day subcutaneously from day 5 to absolute neutrophil count of 1500/mmc for three days.

**Results:**

The median time to absolute neutrophil count engraftment was 11 days for the filgrastim XM02 group and 12 days for the lenograstim group. As for platelets recovery, the median time was 12 days in both groups. The median number of G-CSF vials used for patients was 9.5 for Tevagrastim and 10.5 for lenograstim, reflecting a mean estimated cost of about 556.1 euros for Tevagrastim versus 932.2 euros for lenograstim (*p*< 0.001). The median days of febrile neutropenia were 1.5 and 1 for filgrastim XM02 and lenograstim, respectively. No adverse event related to the use of XM02 filgrastim was recorded.

**Conclusion:**

In our experience, filgrastim XM02 and lenograstim showed comparable efficacy in shortening the period of neutropenia after cytoreduction and autologous stem cell transplantation, with a favourable cost effect for filgrastim XM02.

## Introduction

Granulocyte colony-stimulating factors (G-CSFs), lenograstim and filgrastim, are biological growth factors that promote the proliferation, differentiation, and activation of neutrophils in the bone marrow. Lenograstim is derived from Chinese hamster ovary cells consisting of 174 amino acids with 4% carbohydrate, indistinguishable from native G-CSF, whereas filgrastim is produced in *Escherichia coli*differing from lenograstim in being non-glycosylated and in having an extra methionine group at the N-terminal end of the peptide chain [1–3].

Both are recognised to be useful in the treatment in chemotherapy-induced febrile neutropenia, in peripheral blood stem cells (PBSCs) mobilisation and in promoting recovery after autologous stem cell transplantation [1, 2, 4–8].

The American Society of Clinical Oncology’s 2006 update on the use of white blood cell growth factors provides recommendations for primary and secondary G-CSF prophylaxis [10–12]; they confirm the positive impact of G-CSF in mobilising PBSCs and in accelerating recovery after cytoreduction chemotherapy treatment and PBSC transplantation.

The clinical efficacy of lenograstim and filgrastim has been compared in a systematic review of 16 studies, showing no differences between the two growth factors in any of the approved indications [13].

Biosimilars are non-identical versions of originator biopharmaceuticals. They differ from originator drugs in the size of the active substance, complexity, and nature of the manufacturing process. Several biosimilar G-CSFs are approved in Europe: Biograstim®/Filgrastim, ratiopharm/Ratiograstim®/Tevagrastim® (XM02), Zarzio® and Nivestim®. Recently, biosimilars have been routinely introduced in clinical practice, in particular in the treatment of cancer neutropenia [14]. On the basis of these results, the European Medical Association (EMA) extrapolated the therapeutic equivalence of the biosimilars for PBSC mobilisation and recovery after autologous stem cell transplantation despite the fact that there is little data supporting clinical equivalence. The relevance of biosimilars is mainly related to cost-efficient analysis despite the limited experience at the time of approval of these products in terms of efficacy, safety, and immunogenicity.

Here, we report our experience of the use of G-CSF biosimilar Tevagrastim (filgrastim XM02), compared with branded lenograstim (Myelostim) in the recovery after PBSC transplantation, taking into account their cost-efficacy profile.

## Patients and methods

### Patient characteristics

From November 2010 to December 2011, 26 consecutive patients with haematological disease (13 with non-Hodgkin’s lymphoma, nine with multiple myeloma) and four patients with solid tumours (testicular seminoma) underwent PBSC transplantation in our haematoncology division and received lenograstim after PBSC reinfusion to accelerate engraftment. The median age was 58 (range 18–73). Thirty-two patients with haematological disease (six with Hodgkin’s lymphoma, 13 with non-Hodgkin’s lymphoma, 13 with multiple myeloma), with similar median age (58 years, range 17–76) received G-CSF biosimilar (filgrastim, Tevagrastim) at the same dosage. The conditioning regimen was ICE (ifosfamide, citarabine, etoposide) for testicular seminoma, R-FEAM or R-BEAM (Rituximab, fotemustine or carmustine, etoposide, citarabine, melphalan) for high-grade B cell lymphomas or for Hodgkin lymphoma, Melaphalan for multiple myeloma, and Rituximab–Novantrone–melphalan for follicular lymphomas. Clinical characteristics are reported in Table 1.

All patients received Tevagrastim or lenograstim after PBSC reinfusion at the dosage of 5 mcg/kg/day subcutaneously from day +5 until absolute neutrophil count (ANC) of 1500/mm^3^for three days.

Engraftment was defined as ANC > 500/mmc and platelet count > 20,000/mmc.

Febrile neutropenia was defined as grade 4 neutropenia (ANC<500/mm^3^) with an axillary temperature ≥ 38.5 °C or two or more febrile episodes at > 38 °C within a 12-h period.

The primary data from clinical records were stored in the divisional database containing detailed information of patients hospitalised at the European Institute of Oncology for autologous transplantation and including demographic and diagnostic variables as well as conditioning regimen and data regarding post-transplant complications and haematological recovery.

A cost-effective analysis considering engraftment, incidence of febrile neutropenia, number of days of hospitalisation, and number of vials of G-CSF administered was performed.

## Statistical analysis

The patients’ characteristics have been summarised and tabulated using either counts and percentages for categorical data or count, mean, median standard error, min and max for continuous variables. The median days to reach the ANC cut-off values of 0.5 x 10^9^/L by treatment have been estimated by the Kaplan–Meier method (log-rank test for treatments comparison) and tabulated with 95% confidence intervals. The hazard ratios have been calculated taking lenograstim as reference. All other treatment comparisons were done using the two-sample Wilcoxon test or the unpaired *t*-test as appropriate. All tests were considered statistically significant at the 5% level and two tailed. Statistical analyses were performed using the SAS 9.2 (Cary, North Carolina, United States).

## Results

### Engraftment

The median number of CD34+ cells reinfused was 3.05 x 10^6^/kg (2.0–9.5) and 3.84 x 10^6^/kg (2.0–10.0) in the lenograstim group and filgrastim group, respectively (*p*= 0.437).

The median number of days to ANC recovery was 12 days (10–12) in the lenograstim group and 11 (10–11) in the tevagrastim group without statistically significant difference (*p*= 0.055) (Table 2). Regarding platelets recovery, median days were 12 in both group with a range of 7–22 days for the tevagrastim group and 9–30 days for the lenograstim group.

## Safety

The median number of days of febrile neutropenia was 1 (range 0–10) for the lenograstim group and 1.5 (range 0–5) for the tevagrastim group without statistically significant difference; the median days of antibiotic therapy was 3.5 (range 0–12 ) for the filgrastim group and 4 (range 0–22) for the tevagrastim group (*p*ns). With a median follow-up of 14 months (range 3–23), neither systemic or local side effects nor immunogenicity related to the filgrastim was observed.

## Cost analysis evaluation

The median number of G-CSF vials administered of lenograstim and tevagrastim was, respectively, 10.5 (11.7 ± 0.7) and 9.5 (9.6 ±0.3), with a statistically significant difference (*p*0.027). Performing a cost analysis evaluation, we found a mean estimated cost about of 932.2 ± 54.4 euros for filgrastim and 556.1 ± 20.0 euros for tevagrastim (*p*< 0.001) (Table 3).

## Discussion

Different studies have evaluated the impact of lenograstim in the treatment of chemotherapy-induced febrile neutropenia [15], in peripheral stem cells mobilisation and engraftment [16–18]. In addition, in 2011, Sourgens *et al*[13] performed a systematic review of 16 studies comparing filgrastim with lenograstim. No clinically remarkable differences between filgrastim and lenograstim in chemotherapy-induced neutropenia and mobilisation of peripheral blood progenitor cells in patients and healthy donors were observed. In conclusion, there is no reason to prefer lenograstim over filgrastim in any of the approved indications for both.

Biosimilars are non-identical versions of originator biopharmaceuticals. From their introduction to clinical practice, a cost-efficient impact has been evaluated in different onco-haematological fields.

The first application of biosimilars was the chemotherapy-induced febrile neutropenia. Salesi *et al*[19] reported that the use of biosimilar G-CSF is safe and effective in reducing neutropenic complications in patients with solid tumours and may be associated with cost saving. Also, Aapro *et al*[20] in a comparative cost-efficiency analysis across the European G5 countries of various regimens of filgrastim, biosimilar filgrastim and peg-filgrastim reported that Zarzio® is the most cost-efficient approach in reducing the incidence of febrile neutropenia in chemotherapy-treated patients. Kotwica *et al*[21] in their experience of 23 consecutive patients concluded that biosimilar G-CSF appeared to be effective in reducing the duration of neutropenia in patients undergoing myeloablative therapy followed by autologous Stem cell Transplantation with cost savings in cancer supportive care budgets.

Regarding the application of biosimilars in peripheral stem cells mobilisation, our group reported their experience on the use of Plerixafor in 28 patients treated with the G-CSF originator or with G-CSF biosimilar (Tevagrastim) in patients with lymphoma or myeloma candidate to autologous stem cell transplantation. In this report, no difference was observed with the use of G-CSF or biosimilar Tevagrastim, and no major side effect was reported either with G-CSF or biosimilar Tevagrastim [22].

Finally, the impact of biosimilars was evaluated in the engraftment after PBSC reinfusion. Publicover [23] demonstrated no differences between the originator versus G-CSF biosimilar (ratiopharm) not only in CD34+ mobilisation and harvest but also in median days to obtain neutrophil engraftment (13 ± 2.4 vs 13 ± 2.9 , *p*0.26) and platelet engraftment (12 ± 3.9 vs 12 ± 4.1, *p*0.33), without any increased toxicity with the biosimilar ratiopharm.

Lefrère *et al*[24] enrolled 40 patients affected by haematological malignancy to receive biosimilar G-CSF (Zarzio) after the first cycle of chemotherapy. These patients were compared with a historical control group treated with G-CSF (Neupogen) in the same centre and clinical protocol. No significant differences were observed between groups in median CD34+ cells mobilised and harvested or the number of G-CSF injections to obtain at least 3 x 10^6^CD34+ cells/kg (minimal CD34+ cells count considered successful). The benefit of biosimilar G-CSF in PBSC mobilisation with regard to safety and cost saving was unquestionable due to the identical median consumption of G-CSF doses and leukapheresis procedures noted to reach the target of a minimal count.

Finally, Czerw *et al*[25] showed the results of 35 consecutive patients with multiple myeloma treated with Neupogen and 55 with Zarzio: in this analysis, GCSF biosimilar provides equivalent efficacy in accelerating neutrophil recovery after ASCT compared with the original formulation.

In our analysis, patients with haematological and non-haematological disease have been included. Two different agents have been compared in the post-autologous transplantation: the originator lenograstim, (myelostim) and biosimilar filgrastim (Tevagrastim) used in our institution.

In our experience, tevagrastim and myelostim were both used at 5 mcg/kg/die. In particular, we did not find a significant statistical difference in terms of ANC recovery between filgrastim biosimilar and lenograstim originator. Nevertheless, this result has been obtained with a lower median number of G-CSF biosimilar vials (9.5) than G-CSF originator (10.5) with a statistically significant difference.

Both treatments were safe. With a median follow-up of 14 months we did not observe any deaths related to the autologous procedure.

According to multivariate analysis, no statistically significant differences were observed in the two groups in terms of number of febrile neutropenia; engraftment and days of hospitalisation were similar. Nevertheless, the cost between biosimilar G-CSF and originator is very different, biosimilars being cheaper than G-CSF originators. This could allow a significant cost reduction at our centre.

To the best of our knowledge, this is the first experience comparing lenograstim versus biosimilar filgrastim, showing no differences in activity and toxicity, with a 27% cost savings in favour of biosimilar.

## Disclosures

The authors have declared no conflicts of interest.

## Acknowledgment

The cost of publication was kindly donated by Gruppo Italiano Infermieristico Mobilizzazione e Aferesi (GIIMA).

## Figures and Tables

**Table 1:** Patients’ characteristics.

	FILGRASTIM (biosimilar)	LENOGRASTIM (branded)
**Number of patients**	32	26
**M/F**	20/12	16/10
**Median age y, range**	58.5 (17-76)	58.5 (27-73)
**Disease**		
Non-Hodgkin’s Lymphoma	13	13
Hodgkin’s Lymphoma	6	0
Multiple myeloma	13	9
solid tumours	0	4
**Conditioning regimens**		
R-Novantrone-Melphalan	2	5
Melphalan	15	10
BEAM +/- R or FEAM	15	7
ICE	0	4
**CD34+ reinfused (10^6^/Kg)**		
median number, range	3,84 (2-10)	3,05 (2-9,5)

**Table 2:** Median days to neutrophiles count cut-off values by treatment.

	Treatment	N	Median (95% CI)	Hazard Ratio (95% CI)	P-Value
Days to ANC > 0.5 × 109/L	Lenograstim	26	12 (10,12)	Reference	
	Tevagrastim	32	11 (10,11)	1.78 (1.00,3.14)	0.055

**Table 3:** Multivariate analysis.

	Treatment	N	Mean ± StdErr	Median	P-Value
Age (y)	Lenograstim	26	52.1 ± 3.1	58.5	0.864
	Tevagrastim	32	53.8 ± 2.7	59.5	
CD34+ reinfused (#)	Lenograstim	26	3.81 ± 0.39	3.05	0.437
	Tevagrastim	32	4.05 ± 0.33	3.84	
vials G-CSF administered (#)	Lenograstim	26	11.7 ± 0.7	10.5	***0.027***
	Tevagrastim	32	9.6 ± 0.3	9.5	
Euros (#)	Lenograstim	26	932.2 ± 54.4	833.5	***< .001***
	Tevagrastim	32	556.1 ± 20.0	552.4	
Hospitalisation (days) (#)	Lenograstim	26	16.7 ± 0.7	16.5	0.500
	Tevagrastim	32	16.1 ± 0.6	16.0	
Days of Febrile Neutropenia (#)	Lenograstim	26	2.3 ± 0.6	1.0	0.834
	Tevagrastim	32	1.6 ± 0.3	1.5	
Days of I.V. Antiobiotics (#)	Lenograstim	26	4.0 ± 0.8	3.5	1.000
	Tevagrastim	32	4.3 ± 0.9	4.0	
